# Supplementary material for: Motivations for and experiences of childbirth abroad amongst Nigerian women: A qualitative study
Source: PLOS Glob Public Health. 2024 Sep 13;4(9):e0003737. doi: 10.1371/journal.pgph.0003737 (PMC11398654; doi:10.1371/journal.pgph.0003737)
Supplement: S2 File — (DOCX) [file pgph.0003737.s002.docx]

**Supplementary file 2: Recruitment advert**

**Are you a Nigerian woman above 18 years?**

**Have you gone abroad to have any of babies?**


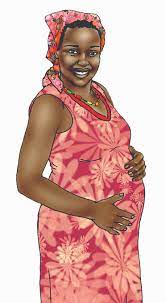

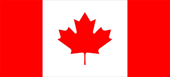

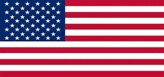

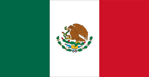

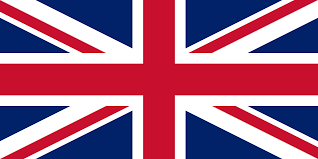

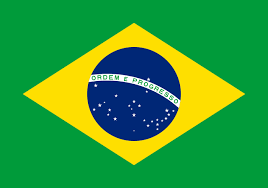


*If you answered* ***YES*** *to both questions, then we would like to invite you for an interview to help us better understand your motivations and experiences of seeking childbirth abroad.*

Insights from your interview will help in shaping future policy and programmes to support women going for childbirth abroad.

Please note that your participation is entirely voluntary, and you may choose to leave the study at any point if you experience discomfort or find that there are any parts of this study that you do not wish to complete.

If you choose to participate, then please complete the informed consent and send via email to:

[a.bankethomas@gre.ac.uk](mailto:a.bankethomas@gre.ac.uk) OR complete it via (link provided to this DocuSign link.

We will be in touch to schedule a convenient time for the interview and send you a N10,000 (£20) mobile phone recharge card upon completion of the interview.
