# Supplementary material for: Motivations for and experiences of childbirth abroad amongst Nigerian women: A qualitative study
Source: PLOS Glob Public Health. 2024 Sep 13;4(9):e0003737. doi: 10.1371/journal.pgph.0003737 (PMC11398654; doi:10.1371/journal.pgph.0003737)
Supplement: S3 File — (DOCX) [file pgph.0003737.s003.docx]

**Supplementary 3: Interview guide**

**Agenda**

Introduction

We are meeting to explore your motivations and experiences of seeking childbirth abroad. What we discuss here will remain confidential. I will start by asking you to describe your family including the number of children you have and your socio-economic status. Then I would ask you to describe why you choose to have your child abroad, your experience in planning the childbirth, the process itself and your experience during childbirth. We will then discuss if you have realised your motivations and whether you think it was worth it. Finally, I will ask you to share your pieces of advice for governments and other women. The entire session will take 40-50 minutes. With your permission, we will record the interview but nothing you say on tape will be specifically linked to you.

*Questions*:

1. Please tell me about your family. Are you married? If yes, when did you marry? How many children do you have?

*(Probe) Please tell me more…*

- 1. Could you kindly provide more detail?
  2. What has the experience of being a mother been like?

1. Where did you deliver your child? And why did you choose to have your child in [Country X]?

*(Probe) Please tell me more…*

- 1. Could you describe to me the process of deciding this country?
  2. What factors are considered in selecting this country?

1. What was the process of securing childbirth in this country like?

*(Probe) Please tell me more…*

1. What was the process of childbirth in the country like?

*(Probe) Please tell me more…*

- 1. Where did you deliver? What was it like? How was the care? How were the nurses, midwives, and doctors?
  2. How was the care different from what you would get in your country?

1. Would you say your motivations for having your child abroad has been realised?

*(Probe) Please tell me more…*

1. What recommendations do you have for other women like you who have their babies abroad?

*(Probe each recommendation, if needed) Please tell me more…*

1. What recommendations do you have for the government as it relates to women like you who have their babies abroad?

*(Probe each recommendation, if needed) Please tell me more…*

1. What else would you like to tell me?

I would like to thank you for your participation.
