# Supplementary material for: Motivations for and experiences of childbirth abroad amongst Nigerian women: A qualitative study
Source: PLOS Glob Public Health. 2024 Sep 13;4(9):e0003737. doi: 10.1371/journal.pgph.0003737 (PMC11398654; doi:10.1371/journal.pgph.0003737)
Supplement: Attachment — Submitted filename: Responses_to_reviewers_PGPH_Final.pdf [file pgph.0003737.s005.pdf]

10th June 2024

Dr Sunday Adedini
Academic Editor
PLOS Global Public Health

Dear Editor,

Thank you very much for considering our paper and for the comments provided by the reviewers, which have helped to significantly improve the quality of the paper.

We have addressed these comments and provided point-by-point response as set out below.

We eagerly look forward to your feedback.

Thank you for your consideration.

Regards,

Dr Aduragbemi Banke-Thomas
Corresponding author
on behalf of all authors

Journal Requirements:
1. Please include a complete copy of PLOS' questionnaire on inclusivity in global research in your revised manuscript. Our policy for research in this area aims to improve transparency in the reporting of research performed outside of researchers' own country or community. The policy applies to researchers who have travelled to a different country to conduct research, research with Indigenous populations or their lands, and research on cultural artefacts. The questionnaire can also be requested at the journal's discretion for any other submissions, even if these conditions are not met. Please find more information on the policy and a link to download a blank copy of the questionnaire here: https://journals.plos.org/globalpublichealth/s/best-practices-in-research-reporting. Please upload a completed version of your questionnaire as Supporting Information when you resubmit your manuscript.

Response: We have completed and submitted this form, as requested.

2. We have noticed that you have uploaded Supporting Information files, but you have not included a list of legends. Please add a full list of legends for your Supporting Information files after the references list.

Response: We have included a list of the supporting information files submitted after the reference list.

Additional Editor Comments (if provided):
As the reviewers have indicated, the manuscript is interesting and focuses on less studied subject. However, the manuscript requires a major revision. The authors are invited to address the concerns raised and resubmit the revised manuscript.

Response: We thank you for taking on the edit of this very important paper. We are enthused by the assessment of the reviewers and glad they agree with us that this is an important yet less studied subject matter. We have addressed all concerns raised and resubmitted an improved paper.

Reviewer #1:
Thank you for giving me the opportunity to review this paper. I think it is interesting, with an interesting subject, that I haven't read anything about before. The manuscript is quite easy to read and well-organized and almost written according to SRQR checklist (see my comments). However, it is not ready for publication and needs quite a lot of editing. It also needs language editing.

Response: We sincerely thank you for appreciating and acknowledging the value of our paper and your kind words regarding it being well organised. We have reviewed it from top to bottom and edited the language as deemed necessary to ensure that is now in a form ready for publication.

Overall, you use deliver and give birth interchangeably in the paper. Women give birth (they do not deliver babies) so please stick to "Nigerian women who have given birth" (or equally), and births instead of deliveries (in the whole paper including table).

Response: Thank you for this comment. We used to "deliver" and "delivery" as these are synonyms of give birth and can communicate childbirth in one word unlike the alternative which could require 1-3 words depending on the specific sentence construction. See link that lists synonyms of "give birth": https://www.thesaurus.com/browse/give-birth. We agree that it might be worthwhile to stick to one or the other. As such to address this comment, we have used your preferred phrase. We have also changed our title to "Motivations for and experiences of childbirth abroad amongst Nigerian women: A qualitative study" (Page 1)

In the introduction some topics can be made clearer for the reader:
- You write "At one airport in the UK, the government claimed that during a two-year period, immigration officials stopped over 300 expectant mothers with pregnancies too advanced to be put back on planes to

fly back home." To me it's not clear whether these mothers are actually birth tourists, from your explanation it seems as though they opted to go back home but couldn't?

Response: Thank you for this comment. We have rephrased this sentence to improve clarity. It now reads, "At one airport in the UK, the government claimed that during a two-year period, immigration officials stopped over 300 expectant mothers with pregnancies which were too advanced making it difficult for officials to put the women back on planes to return to their homes" (Page 3, Paragraph 1). Please note that in the cited article, the women were not specifically labelled "birth tourists" though the article is about birth tourism. See link to the cited article below:
https://www.magazine.medicaltourism.com/article/maternity-tourism-like-stealing-candy-baby

- You also write "More recently, in Canada, a retrospective analysis of 102 women identified through a Central Triage system as women who delivered in Calgary over an 18-month period found that most were from Nigeria (24.5%)." From your explanation I can't see that this is actually about birth tourism.

Response: Thanks for this comment. As the authors of this study assumed that these women were birth tourists, we have now added, "…deemed to be "birth tourists"…" for clarity. (Page 3, Paragraph 1)

- Several sentences are very long (>50 words) and needs to be shortened or split in two.

Response: We have worked through the introduction and indeed the entire manuscript and tried to split long sentences. See multiple examples across the entire manuscript.

- Your aim is to "understand the motivations and experiences of pregnant Nigerian women who travelled out of their country to give birth in another country" and you write that there is only one other study focusing on women's experiences of birth tourism, but no other reflection is made. I really miss info on what those women experienced as your paper is qualitative and about experiences. Your objective could be made clearer like this: Our objective of this study is to explore the motivations and experiences of pregnant Nigerian women who travel out of Nigeria to give birth in another country.

Response: Thank you for this comment. In the introduction and specifically, the key point we wanted to make here is that this subject matter is only minimally studied. We have highlighted our reflection on this being that no study has been on Nigerian women, amongst whom the practice is common. We have also rephrased our study objective as you advised. It now reads, "Our objective in this study is to address the gap by seeking to understand the motivations for and experiences of pregnant Nigerian women who travelled out of Nigeria to give birth in another country" (Page 4, Paragraph 1).

Study setting needs to be proofread. Also, the sentence "As established already, Nigeria is one of the top countries of origin of women who seek childbirth abroad, and indeed, giving birth has been described as a trend in the country in recent years." is missing some word?

Response: Indeed, this is missing a word. We have added "…abroad…". The sentence now reads, "As established already, Nigeria is one of the top countries of origin of women who seek childbirth abroad,2,4 and indeed, giving birth abroad has been described as a trend in the country in recent years.21" (Page 4, Paragraph 3).

Recruitment needs to be further explained, what group do you refer to here: "Through the advert accessed via a web link posted in the group,"

Response: We are referring to the social media groups. We have added this to improve clarity. The statement now reads, "Through the advert accessed via a web link posted in the social media groups…" (Page 5, Paragraph 1).

How is IDIs ideal for maintaining confidentiality? If you are only striving for confidentiality, then I would use an anonymous questionnaire with open ended questions. Wasn't your aim with IDIs to have depth in your data?

Response: Thank you for this comment. We used IDIs as opposed to FGDs which would have meant other women would have met with other women and have confidentiality compromised. To address this comment, we have deleted the sentence altogether.

How was the interview-guide "pre-tested" (pilot tested)?

Response: We have expanded the sentence to reflect how the guide was pilot tested writing, "…using an open-ended interview guide which was pilot tested with women not involved in the actual study" (Page 5, Paragraph 3).

How many interviews did each author perform? How could the women request who was going to perform the interview? In the Author contributions you write that all authors were involved in the interviews but in the methods section only two?

Response: All authors were involved in the interviews. However, each interview was conducted by two interviewers. We have made this clearer by writing, "Each interview was conducted by two members of the team, as requested by the women during recruitment" (Page 6, Paragraph 1). We have also justified our approach writing, "To ensure women felt most free to share during the interviews, they were offered the option to select which two members of the research team they preferred to conduct the interview from a list that described the background of all team members" (Page 5, Paragraph 1).

Were the interviews conducted using video call or only audio? Were videos or only audio recorded?

Response: We already stated in the manuscript that the interviews were audio-recorded (Page 6, Paragraph 2). We also established this in the abstract.

In the methods section you write that collection of data continued until thematic saturation was achieved, however in the discussion you write "However, we continued data collection beyond when data saturation was achieved and continued with additional IDIs as long as we had an opportunity to recruit Nigerian pregnant women to the study." Explain and make this clear in the method section, please.

Response: Thank you for picking this discrepancy. We have aligned both to reflect continuing data collection until thematic saturation was achieved (Page 6, Paragraph 2 and Page 23, Paragraph 2).

I really like that you confirmed your findings with the interviewees, I'd call it "member -checking"

Response: Thank you for this comment. Indeed, this is the technical nomenclature. We decided to keep our more descriptive narrative of what we did in this case (Page 6, Paragraph 2). This lay description is captured in the paper below:
Birt L, Scott S, Cavers D, Campbell C, Walter F. Member Checking: A Tool to Enhance Trustworthiness or Merely a Nod to Validation? Qualitative Health Research. 2016;26(13):1802-1811.

I would strongly recommend you use an additional reference to the Braun and Clarke reference from 2006. They have developed the method a lot since then and published a lot on the developments (as an example they call their method "Reflexive thematic analysis" now).

Response: Thanks for flagging this to us. Much appreciated. We have another reference from Braun and Clarke. See Ref 29. As we have not used this method, as now described "reflexive thematic analysis" in this study, we decided against citing any additional sources.

The analysis need to be further explained, it is very brief now and does not explain the process, how were categories/themes developed, who were involved and so on (see the SRQR checklist)

Response: Thank you for pointing us to this. We have expanded our explanation of our description of our data analysis, explaining the process in granular details, how we coded, and how the themes were developed and refined (Page 6, Paragraph 4).

I don't understand how the sex of the babies are important for the findings? I would delete that result.

Response: The sex of babies has been highlighted in the literature as informing the choice of Chinese Women to seek childbirth abroad. We were keen to see if this is the case amongst Nigerian women also, especially as there is a recognised boy preference in the society.

Fuse K (2008) Cross-National Variation in Attitudinal Measures of Gender Preference for Children: An Examination of Demographic and Health Surveys from 40 Countries. Available at: https://dhsprogram.com/pubs/pdf/WP44/WP44.pdf

We have made this more clear in the manuscript in the methods and discussed the point that we have not observed any different in motivation by sex of babies (Page 23, Paragraph 4).

The themes seem underdeveloped, Braun and Clarke explains it as "theme captures an aspect of patterned meaning in the data and tells the reader something about the shared meaning within it, whereas a topic summary simply summarizes participant's responses relating to a particular topic". To me these themes are topic summaries. I think you can dig deeper into the data and find better themes.

Response: Thank you for this comment. We have reviewed our transcripts again and where necessary draw out patterns in the results. We think this comment might be about style of qualitative research presentation. Our approach was to describe the themes in results and draw patterns while discussing the themes under the Discussion. This is an approach used by many qualitative researchers in published literature. See some recent papers in PLOS Global Public Health:

Baez Caraballo P, Schriger S, Escober J, Acevedo A, García Alejandro A, Halpern M, et al. (2023) Reaching "covidianidad": A qualitative study of the impact of the COVID-19 pandemic on the perceived mental health of health care workers in the Dominican Republic. PLOS Glob Public Health 3(12): e0002652. https://doi.org/10.1371/journal.pgph.0002652

Jennings HM, Anas A, Asmat S, Naz A, Afaq S, Ahmed N, et al. (2024) Living with depression and diabetes: A qualitative study in Bangladesh and Pakistan. PLOS Glob Public Health 4(1): e0002846. https://doi.org/10.1371/journal.pgph.0002846

The quotes often outnumber the describing text in the results which makes me as a reader think the interviews are under-analyzed. As the quotes are often very long and also stacked on top of each other, I recommend you describe more in the text and delete at least half of the quotes (and shorten them).

Response: Thank you for this comment. For such a complex, rarely studied and multi-dimensional topic (country of birth, obstetric history and complication etc.), it is not unusual to have many quotes. It is also important that the narratives of the experiences of women were fully reflected. See example in published literature:

Altshuler AL, Ojanen-Goldsmith A, Blumenthal PD, Freedman LR. A good abortion experience: A qualitative exploration of women's needs and preferences in clinical care. Soc Sci Med. 2017;191:109-116. doi: 10.1016/j.socscimed.2017.09.010.

In response to this comment, we have reviewed all the quotes, deleted some that were redundant and shortened those that we felt their content has been captured elsewhere. Thanks once again. See the results.

I also think the discussion is interesting but very long and a bit wordy. Try to prioritize on what to include there.

Response: We are glad that you found the discussion interesting and agree that the discussion is long, but this is rightly so, as this is such as under-studied topic. We have used the discussion to fully draw upon all the evidence that is available currently and the information in the grey literature. This is the level of attention and detail that this sort of research topic deserves. We carefully chose of journal choice of publication to allow the thorough discussion we believe is merited for our novel research. In response to this comment, we have reviewed the entire discussion and rephrased sentences that came across as wordy to address this comment.

Reviewer #2

Motivations and experiences of Nigerian women who delivered children abroad: A qualitative study

The manuscript examines an important but little-studied topic, the issue of travelling abroad to deliver children among Nigerian women. It provided interesting and relevant information concerning the issue of birth tourism in Nigeria. Having gone through the manuscript, I have the following suggestions to improve its quality.

Response: We thank you for your review and appreciating and acknowledging the value of our work. We have taken up your suggestions to further improve our paper.

Abstract

I think the conclusion section should contain one or two recommendations for action.

Response: We have incorporated recommendations for action in the abstract (Page 2, Paragraph 4).

Introduction

This is adequate as the necessary parts of the introduction can be seen (background, problem and objective).

Response: Thank you for your kind comment. Much appreciated.

Methodology

In the data analysis section, the authors should specify the exact type of thematic analysis used.

Response: Thank you for this comment. We have now specified that we have used a deductive thematic analysis (Page 6, Paragraph 4).

Discussion

The discussion section is adequate. The authors should add a separate conclusion section after the discussion. The paragraph on study strengths and limitations should be given a heading to make it stand out from the rest of the discussion section.

Response: Thank you for this suggestion. We have done this.
